# Video-Assisted Thoracic Surgery vs. Thoracotomy for the Treatment in Patients With Esophageal Leiomyoma: A Systematic Review and Meta-Analysis

**DOI:** 10.3389/fsurg.2021.809253

**Published:** 2022-01-11

**Authors:** Cheng Shen, Jue Li, Guowei Che

**Affiliations:** Department of Thoracic Surgery, West-China Hospital, Sichuan University, Chengdu, China

**Keywords:** thoracotomy, video-assisted thoracic surgery, esophageal leiomyoma, thoracic surgery, meta-analysis

## Abstract

**Background:** Surgical treatment is usually suitable for patients with esophageal leiomyoma. Video-assisted thoracic surgery (VATS) offers a minimally invasive approach to thoracotomy. However, there is no clear conclusion on whether VATS can achieve an equal or even better surgical effect when compared with the traditional open approach in the treatment of esophageal leiomyoma. We performed this meta-analysis to explore and compare the outcomes of VATS vs. thoracotomy for patients with esophageal leiomyoma.

**Methods:** PubMed, Cochrane Library, EMBASE, China National Knowledge Infrastructure (CNKI), Medline, and Web of Science databases were searched for full-text literature citations. The quality of the articles was evaluated using the Newcastle–Ottawa Scale and the data were analyzed using the Review Manager 5.3 software. Fixed or random effect models were applied according to heterogeneity.

**Results:** A total of 8 studies with 290 patients, of whom 141 patients were in the VATS group and 149 in the thoracotomy group, were involved in the analysis. Compared with thoracotomy, VATS was associated with shorter operative time, less blood loss in operation, and shorter postoperative hospital stay. There is no significant difference in postoperative pleural drainage day and postoperative complications between the two groups.

**Conclusions:** VATS has more advantages over thoracotomy, indicating that VATS is better than thoracotomy in terms of postoperative recovery. We look forward to more large-sample, high-quality studies published in the future.

## Introduction

Esophageal leiomyoma is the most common benign tumor in esophageal diseases. The true incidence of esophageal leiomyoma is still uncertain, because many patients with esophageal leiomyoma have no obvious symptoms in clinical practice, and they are often discovered by accident (1, 2). However, despite being the most common benign esophageal neoplasm, it is relatively rare when compared to esophageal carcinoma. Morgagni first described leiomyoma in 1761, but Munro first reported local leiomyoma in the esophageal wall in 1797 (3).

Under normal circumstances, the treatment of these benign esophageal tumors can be removed by surgery. The indications for surgery are large or symptomatic tumors, or tumors that show a growing trend after a period of observation (4). Traditionally, surgical resection is performed through an open method. Ohsawa reported in 1933 that thoracotomy was used to remove esophageal leiomyoma (1). However, minimally invasive methods have gradually replaced traditional methods in recent years (1, 5–7). The surgical option for minimally invasive enucleation of leiomyoma is video-assisted thoracic surgery (VATS) (2, 8, 9).

Although there are several reports on the results of surgical research on esophageal leiomyoma, these studies are of a single center with a small sample size or case reports (6, 7, 10–12), which limits their ability to obtain objective results. Therefore, there is no clear conclusion whether VATS can achieve an equal or even better surgical effect when compared with the traditional open approach in the treatment of esophageal leiomyoma. We performed this meta-analysis to explore and compare the outcomes of VATS vs. thoracotomy for patients with esophageal leiomyoma.

## Methods

### Search Strategy

A systematic literature search was performed in PubMed, Cochrane Library, EMBASE, CNKI, Medline, and Web of Science for studies published before July 2021. The key words used are as follows: “video-assisted OR video-assisted thoracic surgery OR video OR thoracoscopic,” “thoracotomy,” and “esophageal leiomyoma.” Additionally, to avoid duplication of data from different publications from the same author or research team, we further studied these articles to ensure that there was no duplication of research. The inclusion criteria were as follows: (1) clinical studies comparing VATS with thoracotomy in patients with esophageal leiomyoma; (2) full-text articles that reported necessary data for statistical analysis, including at least one of the following outcomes: operation time, estimated blood loss, length of postoperative hospital stay, postoperative duration of drainage, and postoperative complications. The exclusion criteria were as follows: (1) Type of article does not include review articles, case reports, letters to the editor, comments, and meeting reports. (2) Non-human subject studies. (3) Studies without necessary data for statistical analysis. (4) The patients did not undergo surgery. (5) Article not written in English.

### Quality Assessment

The guideline of Newcastle–Ottawa Scale (NOS) was used for evaluating this research including three perspectives of selection, comparability, and exposure. The assessment tool including the star system, a maximum of 9 stars, was used in this research. A specific evaluation system is that 8–9 stars are high quality; 6–7 stars are reasonable quality, and 6 stars less are bad (Table 1).

**Table 1:** Quality assessment of the non-randomized studies using the Newcastle-Ottawa scale.

**References**	**Selection (out of 4)**				**Comparability (out of 2)**	**Outcomes (out of 3)**			**Total score**
	**(1)**	**(2)**	**(3)**	**(4)**		**(5)**	**(6)**	**(7)**	
Xu et al. (2)	1	1	1	1	2	1	1	–	8
Ziyade et al. (1)	1	1	1	1	2	1	1		8
Ramos et al. (13)	1	1	1	1	2	1	–	1	8
Yalçinkaya et al. (14)	1	1	1	1	2	1	–	–	7
Shin et al. (15)	1	1	1	1	2	1	1	–	8
Priego et al. (3)	1	1	1	1	2	1	–	–	7
von Rahden et al. (16)	1	1	1	1	2	1	–	–	7
Wang et al. (17)	1	1	1	1	2	1	1	–	8

*(1) Representativeness of the exposed cohort, (2) selection of the non-exposed cohort, (3) ascertainment of exposure, (4) demonstration that outcome of interest was not present at the start of the study, (5) assessment of outcome, (6) was follow-up long enough for outcomes to occur, (7) adequacy of follow-up*.

### Data Collection

Two reviewers collected data from each study. Any unclear or inconsistent issues are dealt with through discussion. Excel is used to collect the following information (Table 2): author, publication year, country, study design, study period, the sample size in two groups, mean age, gender, tumor size and location, operative time, estimated blood loss, length of postoperative hospital stay, postoperative duration of drainage, and postoperative complications.

**Table 2:** Characteristics of the included studies.

**References**	**Country**	**Design**	**Study period**	**Group**	**Cases**	**Sex** **(M/F)**	**Age**	**Tumor size** (cm)*	Tumor location**		
									**Upper** **third**	**Middle** **third**	**Lower** **third**
Xu et al. (2)	China	R	2008–2017	Thoracotomy	40	26/14	47.23 ± 10.30	3.63 ± 2.15	4	25	11
				VATS	16	11/5	50.06 ± 8.86	2.23 ± 1.30	1	10	5
Ziyade et al. (1)	Turkey	R	1991–2011	Thoracotomy	10	–	49.0 ± 3.02	3.81 ± 2.05	0	1	7
				VATS	8	–	47.6 ± 2.7	4.13 ± 1.68	1	3	6
Ramos et al. (13)	Spain	R	1986–2014	Thoracotomy	7	4/3	51.71 (35–70)	5.48 ± 1.00	0	3	4
				VATS	6	4/2	55.83 (46–70)	4.28 ± 0.50	1	3	2
Yalçinkaya et al. (14)	Turkey	R	2007–2019	Thoracotomy	8	–	–	–	–	–	–
				VATS	5	–	–	–	–	–	–
Shin et al. (15)	USA	R	1995–2011	Thoracotomy	16	–	–	6.40 ± 0.38	3	8	5
				VATS	63	–	–	4.04 ± 0.37	9	32	22
Priego et al. (3)	Spain	R	1986–2004	Thoracotomy	3	–	–	–	–	–	–
				VATS	6	–	–	–	–	–	–
von Rahden et al. (16)	Germany	R	1995–2003	Thoracotomy	12	–	44 (19–63)	–	–	–	–
				VATS	13	–	46 (17–67)	–	–	–	–
Wang et al. (17)	China	R	2005–2013	Thoracotomy	53	–	49.80 ± 4.51	8.60 ± 3.45	10	18	25
				VATS	24	–	38.31 ± 1.90	2.40 ± 0.50	6	8	10

*R, retrospective study; VATS, video assisted thoracic surgery; M, male; F, female; –, not available*.

* *Statistical result was at a P = 0.004 between two groups*.

** *Statistical result was at a P > 0.05 between two groups*.

### Statistical Analysis

All statistical analyses were performed using the Review Manager 5.3 software (Cochrane Collaboration, Oxford, United Kingdom). The dichotomous variables were assessed by using odds ratios (OR) with a 95% confidence interval (CI) and the continuous variables using weighted mean difference (WMD) with a 95% CI. The I^2^ statistics were used to evaluate the heterogeneity. *I*^2^ <25%, 25% ≤ *I*^2^ ≤ 50% and *I*^2^ > 50% were considered to be low, moderate, and high heterogeneity, respectively. If the test of heterogeneity was high (*I*^2^ > 50% or *P* < 0.05), a random-effect model was adopted. Otherwise, we used a fixed effect model. The potential publication bias was evaluated by visually inspecting the funnel plots. *P* < 0.05 was regarded as statistically significant.

## Results

### The Selection of Included Studies

**Figure 1:**
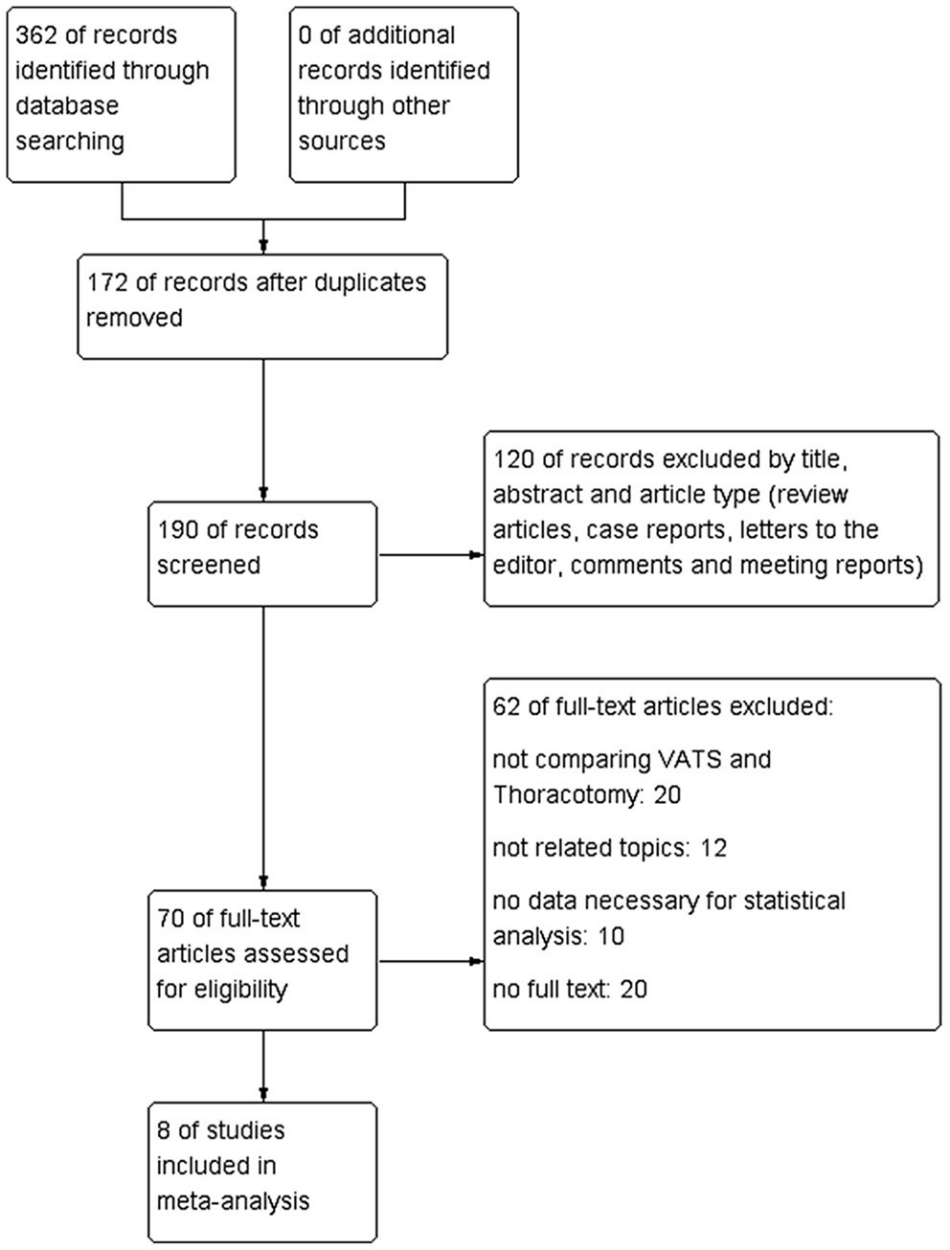
Flow chart of literature search strategies.

We searched four electronic databases including the PubMed, Cochrane Library, EMBASE, CNKI, Medline, and Web of Science and the total number of studies was 362 before June 2021. After duplicates were removed, 190 articles were evaluated carefully. One hundred twenty studies were excluded because they were review articles, case reports, animal experimental studies, letters, meeting abstracts, comments, and other non-related studies. Later, 70 potential articles were further assessed through reading the full texts and there were 62 articles excluded due to inclusion and exclusion criteria. In our meta-analysis, 8 retrospective qualified articles were included finally (Figure 1).

### The Characteristics of Included Studies

A total of 8 studies with 290 patients, of whom 141 patients were in the VATS group and 149 in the thoracotomy group, were involved in the analysis. The basic characteristics of 8 qualified literature sources are recorded in Table 2. Briefly, the mean patient age ranged from 19 to 70 years in the thoracotomy group and 17–70 years in the VATS group. Five studies reported tumor size between the two groups. The statistical result was at a *P* = 0.004 between the two groups, which gives a potential bias between VATS and thoracotomy. These research studies also described the tumor location and most tumors were located in the middle and lower esophagus. There was no significant difference between the VATS group and the thoracotomy group (*P* > 0.05).

Xu et al. (2) revealed short-term clinical outcomes in patients undergoing VATS or thoracotomy. Ziyade et al. (1) enrolled 18 patients and analyzed the outcomes of 8 patients with VATS and 10 patients with open approach. Ramos et al. (13) summarized the perioperative outcomes in patients with thoracotomy and VATS. In Yalçinkaya et al. study (14), VATS was performed on 5 patients, and thoracotomy was performed on 8 patients. Shin et al. (15) evaluated the difference between the open approach and VATS. Priego et al. (3) reported that patients with esophageal leiomyoma underwent surgery *via* open approach or by VATS. von Rahden et al. (16) evaluated the short-term outcomes of 25 patients who underwent surgery for esophageal leiomyoma. The clinical data of the VATS group patients were compared with the data of thoracotomy group patients in Wang et al.'s report (17).

### Meta-Analysis Results

#### The Operative Time Between the Two Groups

**Figure 2:**
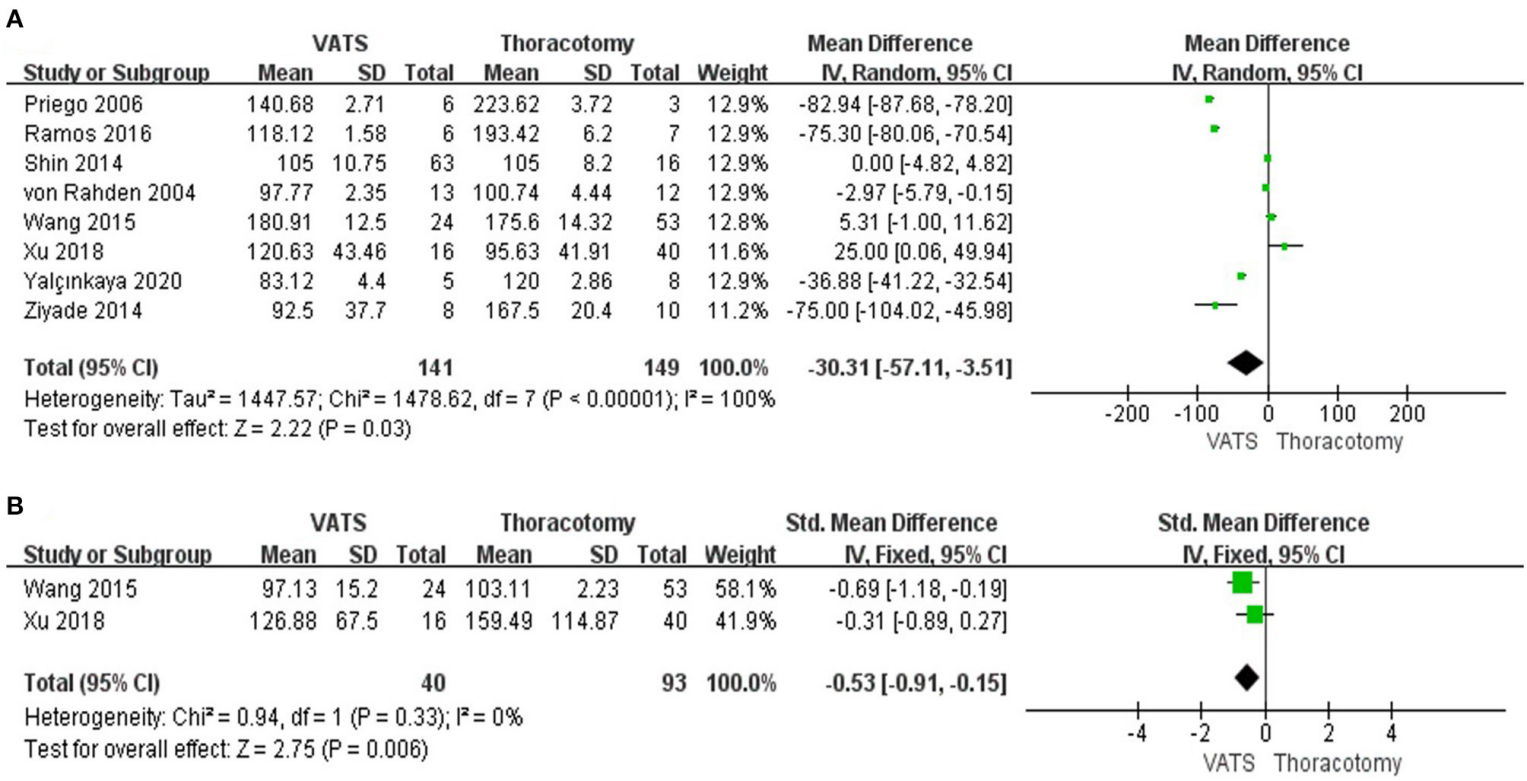
Forest plot of the meta-analysis. **(A)** Operation time. **(B)** Estimated blood loss.

Operation time was reported in all studies. The pooled data revealed that the operative time in VATS was shorter than in open approach (WMD = −30.31, 95% CI −57.11 to −3.51, *P* = 0.03, *I*^2^ = 100%) (Figure 2A).

#### The Estimated Blood Loss During the Operation Between the Two Groups

The data regarding the estimated blood loss were reported in 2 studies. The result showed that the estimated blood loss in the minimally invasive approach was lower than that in the open-access one (WMD = −0.53, 95% CI −0.91 to −0.15, *P* = 0.006, *I*^2^ = 0%) (Figure 2B).

#### The Postoperative Pleural Drainage Days Between the Two Groups

**Figure 3:**
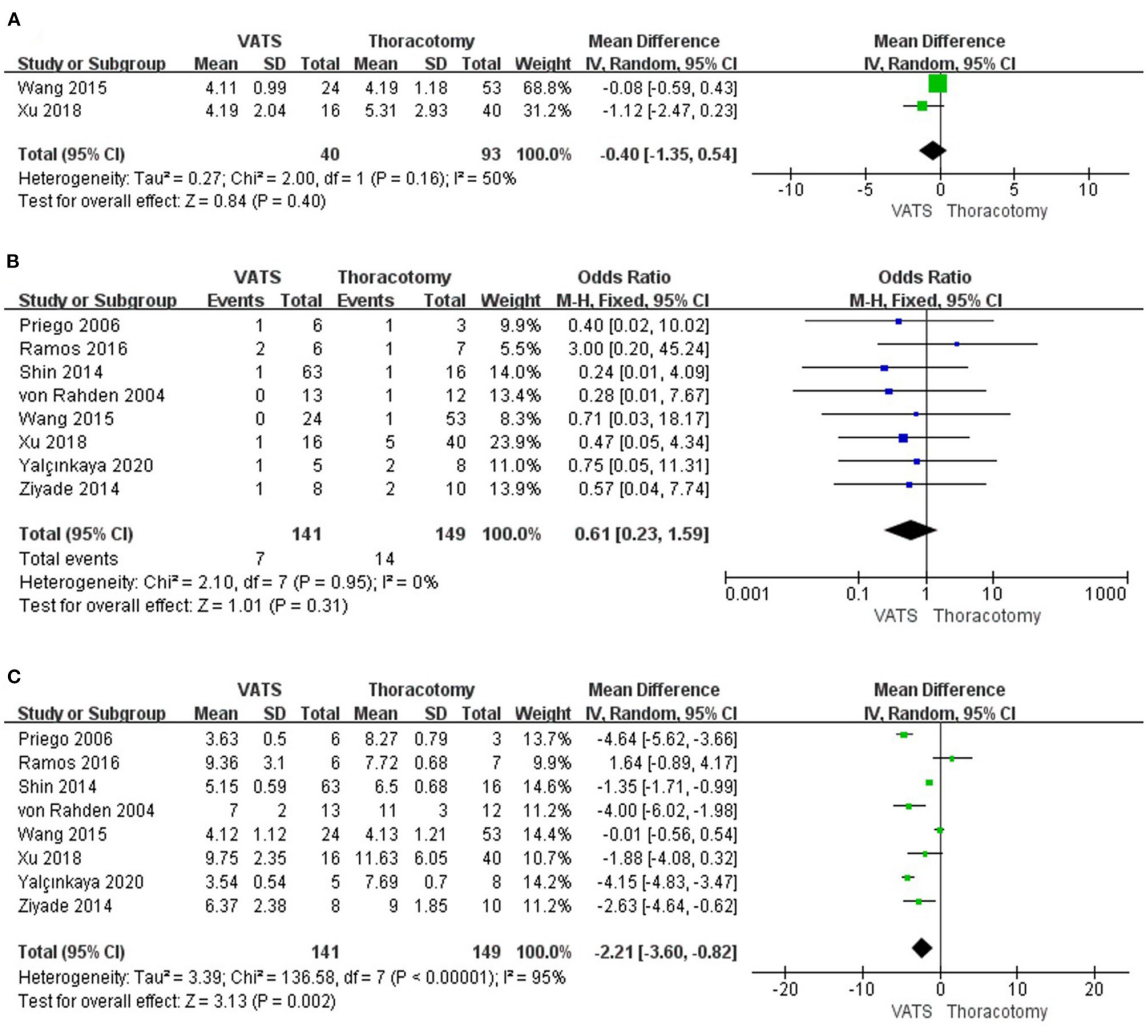
Forest plot of the meta-analysis. **(A)** Postoperative pleural drainage days. **(B)** Postoperative complications. **(C)** Length of postoperative hospital stay.

The pooled results of the 2 studies revealed that there was no significant difference between the VATS group and the thoracotomy group in postoperative pleural drainage days (WMD = −0.40, 95% CI −1.35 to 0.54, *P* = 0.40, *I*^2^ = 50%) (Figure 3A).

#### The Postoperative Complications Between the Two Groups

According to the results of all the studies on overall postoperative complications, no significant results were found between the VATS group and the thoracotomy group (OR = 0.61, 95% CI 0.23–1.59, *P* = 0.31, *I*^2^ = 0%) (Figure 3B).

#### The Length of Postoperative Hospital Stay Between the Two Groups

Results of 8 studies showed that the length of postoperative hospital stay was shorter in the minimally invasive approach group than that in the open group (WMD = −2.21, 95% CI −3.60 to −0.82, *P* = 0.002, *I*^2^ = 95%) (Figure 3C).

## Discussion

Surgical treatment for esophageal leiomyoma is usually suitable for patients with symptoms and tumor is larger than 2 cm (18–22). Thoracotomy is usually used to achieve the purpose of lesion resection, which is regarded as a gold standard surgical approach of treatment for patients at an early stage (19, 20). However, the development of minimally invasive surgery has provided a new method for the treatment of this type of tumor. As shown in our results, minimally invasive treatment options can be associated with shorter operative time, less blood loss in operation, and shorter postoperative hospital stay. Many studies have separately studied the safety and short-term or long-term efficacy of thoracotomy or VATS in esophageal leiomyoma (23), but there is no meta-analysis on the two comparative surgical methods in this disease. We included 8 studies and conducted a meta-analysis to explore and compare the clinical efficacy of VATS and thoracotomy in patients with leiomyoma.

First, the result of meta-analysis in operative time showed that in the VATS group, it was shorter than in the open approach. The main reason might be that the traditional standard posterolateral thoracotomy is complicated. However, it is good for the exposure of the esophagus. Incision and peeling of esophageal leiomyoma is relatively easy during surgery, but a large incision needs to cut part of the chest and back muscles (7). As a result, there are many complications such as severe surgical damage and slow recovery of patients, incision infection, pain, limited upper limb function of surgical side, and respiratory infection (24). It is a typical “small surgery with large incision.” On the contrary, minimally invasive surgery has the characteristics of less trauma, less intraoperative bleeding, quick postoperative recovery, less pain, getting out of bed early, and improving the quality of life of patients after surgery. The advantages of VATS have been accepted by many thoracic surgeons (22).

Regarding the blood loss in operation, our meta-analysis data showed that the intraoperative blood loss was lower in the VATS group than in the thoracotomy group (*P* = 0.006). The main reason is the reduction of intraoperative trauma caused by minimally invasive surgery. During the operation, only 3 small incisions < 2 cm are needed in the chest wall, which can replace the traditional 30 cm incision and the final surgical effect is the same. Another reason might come from the difference in the experience of surgeons. VATS in some medical centers was relatively late and the number of related operations performed by surgeons was small. Most of the cases were still finished in the rising stage of the surgeon's learning curve, which might contribute to longer operative time. In addition, there are fewer patients with esophageal leiomyoma compared to esophageal cancer, and surgical resection methods for them are not the same. So it will prolong the doctor's learning curve. In our included studies, some surgeons had proficient operation experience in esophageal leiomyoma resection by VATS, which made the learning curve for VATS shorter and shortened the operative time significantly. So it is better to control the bleeding of small blood vessels during surgery.

There is no difference in postoperative pleural drainage days between the VATS group and the thoracotomy group. We analyzed the research results and considered that the reason is that the VATS makes the surgical process more delicate, more thoroughly hemostatic, and less stimulating the surrounding tissues. At the same time that the lesion is completely resected and the surrounding adipose tissue can also be completely removed. Eventually, postoperative pleural effusion can be reduced, and postoperative drainage time can be saved.

In the terms of duration of postoperative hospital stay, the result of our meta-analysis revealed that patients in the VATS group were shorter than those in the thoracotomy group (*P* = 0.002). This result is closely related to the application of minimally invasive surgery, which has potential advantages, including less postoperative pain, faster recovery, and a better cosmetic outcome. Enhanced recovery after surgery (ERAS) also showed a significant impact on patients treated with a minimally invasive surgical approach (25). ERAS is the result of the development of medical theory and surgical technology and not only does it pay more attention to reducing patient stress response but also takes into account assessment and intervention of the surgical conduct risk (26–28). ERAS is a series of optimization measures using perioperative management with evidence-based medical evidence to reduce the physiological and psychological traumatic stress to surgical patients to achieve rapid rehabilitation (26, 29). In addition, patients recovered faster after surgery, which also shortened the postoperative hospital stay to a certain extent (30, 31).

Traditionally, the location of the lesion on the esophagus also determines different surgical methods. In esophageal cancer, the right thoracic approach can be used when the cancer is located in the upper or middle segment of the esophagus, and the left thoracic approach can be adopted when the cancer is located in the lower esophagus. Esophageal leiomyoma is a benign tumor, and only needs to incise the muscle layer on the tumor surface for blunt dissection. Some studies have suggested that due to the inverted image of the thoracoscopy, VATS is more complicated when the tumor is located in the lower thoracic segment near the abdomen (14, 23). According to our research, there is no statistically significant difference between VATS and the open approach in terms of the curative effect of tumor location. Minimally invasive surgery can not only satisfy the exposure of the surgical field, but also reduce surgical trauma and postoperative complications, and make the treatment effect better. The imaging technology of VATS can provide a clearer vision, flexibility, and stability of surgical operations, which makes minimally invasive technology a new level.

However, according to Table 2, there is a statistically significant difference between VATS and thoracotomy in terms of the curative effect of tumor size, which gives a potential bias between VATS and thoracotomy. There are several reasons for the result. First, we reviewed the 8 included articles again and found that most of the articles are summarized very early. Compared with the popularity of thoracoscopy, most of them are done by the open approach. Second, the traditional view believes that if a tumor is larger than 5 cm in diameter, it will be difficult to ensure the integrity of the mucosa, and it will be easy to form esophageal diverticula and fistulas or stenosis. Therefore, thoracotomy and gastroesophageal anastomosis should be performed in time when the tumor is larger than 5 cm. Third, in most of the leiomyoma, an enucleation was sufficient over resection and anastomosis and reduced the complexity of the esophageal surgical procedure, which is a huge difference as well.

The occurrence of postoperative complications is also an important indicator for evaluating short-term results after surgery. The complications after esophageal leiomyoma resection are esophageal mucosal injury, intraoperative wound bleeding, mucosal tears, extensive muscle lacerations and defects, postoperative esophageal diverticulum formation, and so on. The pooled results of our meta-analysis showed that there is no significant statistical difference in postoperative complications in the VATS group and the open approach group. The occurrence of this result may be related to several reasons. First, the number of samples included in this study is small, which is not enough to fully reflect the characteristics of postoperative complications. Second, minimally invasive surgery of the esophagus, especially for esophageal cancer, has obvious significance in reducing postoperative complications than open thoracic surgery. The resection of esophageal cancer requires a complete lymph nodes dissection, but esophageal leiomyoma does not require the lymph nodes dissection. Complete lymph nodes dissection will bring more wounds and the possibility of bleeding, or the occurrence of chylothorax.

There are several limitations to this meta-analysis. First, the number of studies included and the simple scale were relatively small. All studies included for meta-analysis were retrospective observational studies and lacked high-quality randomized controlled trials, with a greater risk of potential selection and publication bias. Second, the operative time, blood loss, and length of postoperative hospital stay had significant heterogeneity. Potential factors that could explain the heterogeneity included the different experiences of surgeons and the shorter learning curve for the VATS group.

## Publication Bias

**Figure 4:**
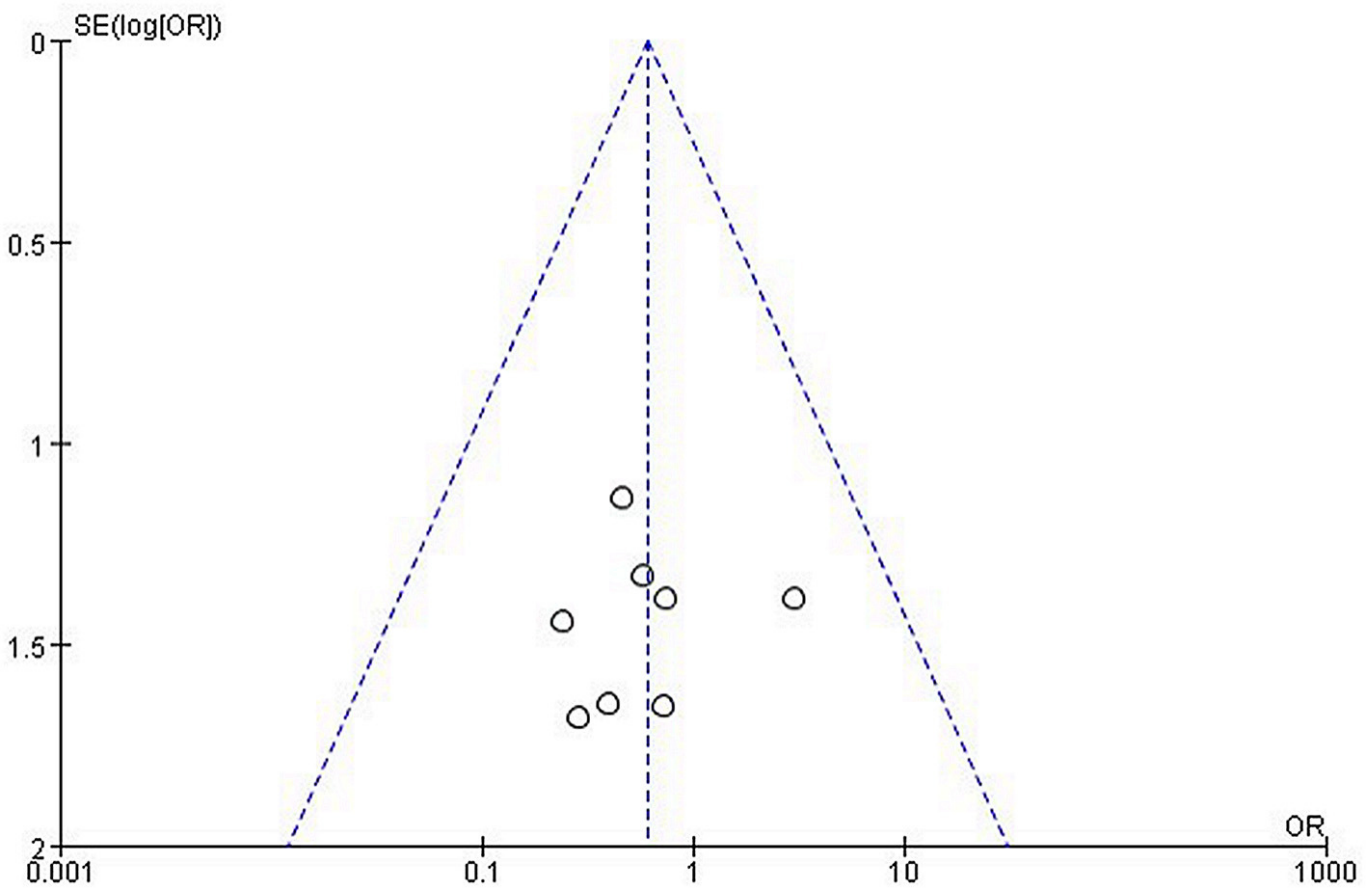
Funnel plot of the meta-analysis.

A funnel plot of the overall complication was used to assess publication bias. The bilaterally symmetrical funnel plot of overall complication showed that no obvious evidence of publication bias was observed (Figure 4).

## Conclusions

In summary, VATS has more advantages over thoracotomy in terms of blood loss, length of postoperative hospital stay, and operative time, indicating that VATS is better than open approach in terms of postoperative recovery. There was no statistically significant difference in postoperative complications and tumor location, indicating that VATS has the same safety and effectiveness as thoracotomy and we can perform minimally invasive surgery on leiomyoma that occurs in all parts of the esophagus. There is currently a lack of long-term follow-up studies for patients after surgery. We look forward to more large-sample, high-quality studies published in the future.

## Data Availability Statement

The raw data supporting the conclusions of this article will be made available by the authors, without undue reservation.

## Author Contributions

CS was involved in drafting the manuscript. JL was involved in the acquisition of data. GC designed and revised the manuscript. All authors have read and approved the final manuscript.

## Funding

This work was supported by the Sichuan Province Science and Technology Support Program (No. 2020JDKP0023) and the Chengdu Science and Technology Support Program (No. 2019-YFYF-00090-SN).

## Conflict of Interest

The authors declare that the research was conducted in the absence of any commercial or financial relationships that could be construed as a potential conflict of interest.

## Publisher's Note

All claims expressed in this article are solely those of the authors and do not necessarily represent those of their affiliated organizations, or those of the publisher, the editors and the reviewers. Any product that may be evaluated in this article, or claim that may be made by its manufacturer, is not guaranteed or endorsed by the publisher.
